# Increased unsaturation of lipids in cytoplasmic lipid droplets in DAOY cancer cells in response to cisplatin treatment

**DOI:** 10.1007/s11306-012-0483-8

**Published:** 2012-12-14

**Authors:** Xiaoyan Pan, Martin Wilson, Carmel McConville, Theodoros N. Arvanitis, Julian L. Griffin, Risto A. Kauppinen, Andrew C. Peet

**Affiliations:** 1 Cancer Sciences, University of Birmingham, Birmingham, NH UK; 2 Birmingham Children’s Hospital NHS Foundation Trust, Birmingham, NH UK; 3 Electronic, Electrical and Computer Engineering, University of Birmingham, Birmingham, NH UK; 4 Department of Biochemistry and the Cambridge Systems Biology Centre, University of Cambridge, Cambridge, NH UK; 5 Clinical Research and Imaging Centre and Department of Experimental Psychology, University of Bristol, Bristol, NH UK; 6 Institute of Child Health, Whittall Street, Birmingham, B4 6NH NH UK

**Keywords:** Lipid droplets, ^1^H NMR, Isolation, Cisplatin

## Abstract

Increases in ^1^H nuclear magnetic resonance spectroscopy (NMR) visible lipids are a well-documented sign of treatment response in cancers. Lipids in cytoplasmic lipid droplets (LDs) are the main contributors to the NMR lipid signals. Two human primitive neuroectodermal tumour cell lines with different sensitivities to cisplatin treatment were studied. Increases in NMR visible saturated and unsaturated lipids in cisplatin treated DAOY cells were associated with the accumulation of LDs prior to DNA fragmentation due to apoptosis. An increase in unsaturated fatty acids (UFAs) was detected in isolated LDs from DAOY cells, in contrast to a slight decrease in UFAs in lipid extracts from whole cells. Oleic acid and linoleic acid were identified as the accumulating UFAs in LDs by heteronuclear single quantum coherence spectroscopy (HSQC). ^1^H NMR lipids in non-responding PFSK-1 cells were unchanged by exposure to 10 μM cisplatin. These findings support the potential of NMR detectable UFAs to serve as a non-invasive marker of tumour cell response to treatment.

## Introduction

An increase in lipids detected by ^1^H NMR has been reported in successful treatment of brain tumours (Griffin et al. 2003; Hakumaki et al. 1999), lymphomas (Schmitz et al. 2005) and breast cancers (Musacchio et al. 2009) indicating their potential as non-invasive biomarkers in cancer management.

Studies on cancer cells in vitro (Shih et al. 2005) and in preclinical tumour models (Griffin et al. 2003) have demonstrated that apoptosis induced by anti-cancer therapies results in subtle increases in saturated aliphatic ^1^H NMR signals as well as unsaturated bis-allylic and vinyl peaks. The increase in ^1^H NMR lipids is closely associated with an increase in size and number of cytoplasmic lipid droplets (LDs) (Mirbahai et al. 2012), the principal source of NMR detectable lipids in vivo (Quintero et al. 2007). While some cells show an increase in the CH_2_/CH_3_ ratio during apoptosis, which could reflect an increase in the proportion of long chain fatty acyl moieties (Shih et al. 2005), other cell lines show no change in this ratio with treatment (Schmitz et al. 2005). Contributions to the –CH_3_ resonance from macromolecules rather than an increase in fatty acid chain length can explain the increased CH_2_/CH_3_ ratio (Mirbahai et al. 2012) but this has not been demonstrated directly in lipid droplets. It has been proposed that NMR visible lipids accumulating during apoptosis originate from membranous cell structures due to lipolysis and repartitioning (Griffin et al. 2003). However, a recent cell study concluded that de novo lipid synthesis may also contribute to the accumulation of ^1^H NMR lipid signals (Boren and Brindle 2012).

We have recently reported that lipids in LDs from neuroblastoma cells contain phospholipids, triglyceride, cholesterol and cholesteryl esters with both saturated and unsaturated fatty acid chains being present (Pan et al. 2012). In the current study, two human primitive neuroectodermal tumour (PNET) cell lines were treated with cisplatin to examine lipid species in whole cells as well as in extracts from whole cell and isolated LD preparations. 1D ^1^H NMR and 2D HSQC were used to characterise the chemical nature of lipid species in untreated and cisplatin treated cancer cells. Our hypothesis was that during cell death, more unsaturated fatty acids would be observed in the ^1^H NMR spectra of whole cells and that this would be reflected in the composition of the isolated lipid droplets. Information on the chemical nature of lipids in LDs will help to improve our understanding of these important structures and how they alter in the cell death process.

## Materials and methods

### Cell culture

Two neuronal brain tumour cell lines: DAOY, human medulloblastoma and PFSK-1, human supratentorial primitive neuroectodermal tumour were cultured in 75 cm^2^ flasks with filter-vented caps and maintained in 15 ml Dulbecco’s modified eagles medium (DMEM F:12) (Invitrogen, UK) supplemented with 10 % (v/v) foetal calf serum (PAA, UK), 1 % 200 mM l-glutamine (100×) and 1 % non-essential amino acid solution (100×) (Sigma, UK). Cells were incubated at 37 °C in a humidified atmosphere (5 % CO_2_, 95 % air).

### Cisplatin exposure

2 mM stock solution was made from cisplatin powder (Sigma Aldrich, UK) with DMEM stored at −80 °C in dark conditions. 10–100 μM cisplatin working solution was freshly made from the stock cisplatin with culture media before adding to the cell culture.

### Alamar blue assay for cell viability

Cells were seeded in flat-bottomed IWAKI 96-well culture plates at a density of 1 × 10^4^cells/well and cultured for 24 h giving a 50–70 % confluence. Cisplatin was added into each well and the plates were incubated at 37 °C and 5 % CO_2_ for 48 h. Alamar blue assay to estimate number of viable cells was performed as previously described (6).

### Isolation of LDs

A protocol developed by Weller et al. was used to isolate lipid droplets (Weller et al. 1991). Briefly, 40–60 × 10^6^ cells were ground in 600 μl deionised water or D_2_O using a Dounce grinder and the homogenate was centrifuged at 2000×*g* at 4 °C for 10 min. The supernatant was adjusted to 18.46 % sucrose, topped with the same volume of deionised water or D_2_O and centrifuged at 142,000×*g* for 120 min at 4 °C (Optima TLX Ultracentrifuge, Beckman). After ultracentrifugation, the sample was separated into three fractions, the upper isolated fraction, the middle sucrose fraction and the pellet. At least three independent preparations for each condition and cell type were used for isolation.

### Lipid extraction

A dual-phase methanol–chloroform extraction protocol was used to prepare the lipid extracts for NMR analysis from both cells and isolated lipid droplets. 20–40 × 10^6^ cells were ground and sonicated in methanol/deionised water. 200 μl chloroform was added to the homogenates twice to form a dual-phase. For isolated LDs, methanol and chloroform was added directly into the isolated fraction. At least three independent preparations from each condition and cell type were used for extraction.

### Nile red and DAPI staining

Cells were spun onto slides and stained with 4 μg/ml Nile red, a dye staining for lipid droplets, in phosphate buffered saline (PBS, made from 1 mg/ml Nile red stock solution in acetone, Sigma Aldrich, Dorset, UK) for about 15 min under dark conditions. After Nile red staining, cells were stained with 0.4 μg/ml DAPI, a dye staining for cell nuclei, for 15 min. 5 μl isolated fraction were dropped on to an ethanol and deionised water washed slide and stained with 4 μg/ml Nile red in 70 % ethanol for 15 min. The slides were visualized with a Nikon Eclipse E600 microscope and images were taken using a DXM1200 digital camera. The green fluorescence of Nile red was observed with FITC (B–2A) filter set, with excitation wave length of 465–495 nm and emission wave length of 550 nm. LD sizes were measured using the image analysis software Image J (National Institute of Health, USA). DAPI UV (UV–2A) filter with excitation wavelength of 340–380 nm was used to detect DAPI stained nuclei.

### HR-MAS

Prior to HR-MAS, frozen cells in PBS were defrosted, and 45 μL of suspension containing approximately 10 × 10^6^ cells was pipetted into a 50 μL rotor (Bruker, Karlsruhe, Germany); 5 μL 50 mmol/L trimethylsilylproprionate d4 (TMS) in D2O was added as a chemical shift standard. HR-MAS was performed on a Bruker 500 MHz spectrometer using a HR-MAS probe (Bruker, Karlsruhe, Germany). The probe temperature was set to 278 K to minimize the degradation. The rotor speed was 4,800 Hz in all experiments. The pulse sequence consisted of a single 90° pulse with one second water presaturation pulse. The receiver bandwidth was 7,200 Hz covered with 16 K complex points. A total of 256 scans were acquired with a repetition time of 3.3 s.

### Liquid-state ^1^H NMR spectroscopy

Lipid extracts were resuspended in 600 μl deuterated chloroform containing 0.03 % (v/v) TMS (Sigma Aldrich, Dorset, UK). ^1^H NMR spectra of cell extracts and the isolated LDs suspended in D_2_O were recorded on a Varian 600 MHz vertical bore spectrometer using a triple-resonance HCN probe. A standard pulse-acquire sequence was used as above for HR-MAS.

### HSQC

A phase-sensitive gradient enhanced 2D ^1^H–^13^C HSQC was performed on whole cell pellets of DAOY cells s on a Bruker DRX 500 MHz spectrometers using the HR-MAS probe at 278 K. 1,024 points were acquired in F2 for 256 increments in F1 with spectral widths of 13 and 166 ppm respectively. 96 averages were performed resulting in a total experiment time of approximately 11.6 h. Data were zero-filled to twice the original length and multiplied by a sine function prior to 2D Fourier transformation. Three independent cell samples were used for HSQC experiment.

### Spectral analysis

The HR-MAS spectra were manually phased and referenced to creatine (3.03 ppm). Baseline correction (Xi and Rocke 2008) was performed and spectra were normalized to the total intensity of the spectral region from 0.5 to 4.5 ppm using in house software written in R. The signal intensity of the lipid peaks around 0.9, 1.3, 2.8, 5.4 ppm and the macromolecular peak at 1.68 ppm were measured using peak integration to estimate the relative abundance of lipid and macromolecule. Values shown are mean ± SD.

For extracts, the spectra were manually phased, then referenced to TMS (0 ppm). The extract spectra were baseline corrected with spectral analysis software, wxNUTs (Acorn NMR Ltd, Canada). The lipid signal intensities were determined using the line fitting function provided by wxNUTs which fits a series of peaks to the experimental spectrum and determines the signal intensity from the fit. Student’s *t* test was performed on the treated versus untreated cell data.

The HSQC spectra were manually phased, referenced to TMS peak at 0 ppm and integrated with Topspin 3.0 software (Bruker, Germany).

## Results

The Alamar blue assay showed that with a 48 h exposure to 10 μM cisplatin, 5 ± 0.2 % of DAOY cells were viable while around 60 ± 1.1 % of PFSK-1 cells were still viable. DAOY showed a clear response to 10 μM cisplatin, whereas PFSK-1 did not. Thus, because of the discrimination, this concentration with a 48 h exposure time was chosen for subsequent experiments.

**Fig. 1:**
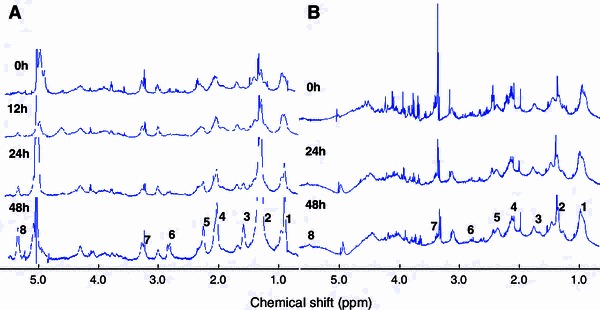
HR-MAS spectra of DAOY (**a**) and PFSK-1(**b**) cells treated with 10 μM cisplatin at 0, 12, 24 and 48 h. Assignments are as follows: 1 C**H**_3_ at 0.9 ppm, 2 C**H**_2_ at 1.2–1.3 ppm, 3 C**H**_2_CH_2_CO at 1.6 ppm, 4 C**H**_2_CH=CH at 2.0 ppm, 5 C**H**_2_COO at 2.3 ppm, 6=CHC**H**_2_CH= at 2.8 ppm, 7 N(C**H**_3_)_3_ at 3.3–3.4 ppm, 8 **H**C=C**H** (L, Ch) at 5.4 ppm

Figure 1 shows the HR-MAS spectra of DAOY cells treated with cisplatin, the spectra were scaled to the height of the macromolecule peak at 1.68 ppm for display. For quantification, the lipid signal intensities were taken as a ratio to the macromolecule peak area at 1.68 ppm as referencing to protein signal has been shown to be a good approach in NMR signal quantification **(**Bayet-Robert et al. 2010) and renders the data from different cell lines comparable.

**Fig. 2:**
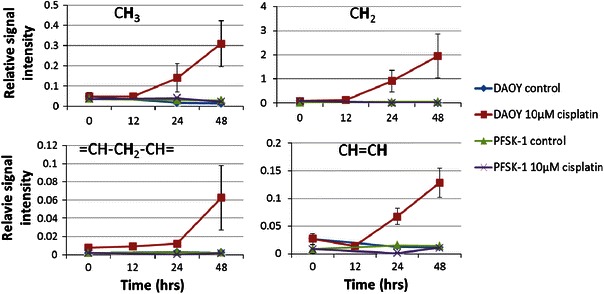
Lipid signal intensity of lipid peaks at 0.9, 1.3, 2.8, 5.4 ppm of DAOY and PFSK-1 cells with or without 10 μM cisplatin exposure. Values were obtained from HR-MAS spectra of intact cells

Lipid signal intensities in HR-MAS spectra of DAOY and PFSK-1 cells, as a function of cisplatin exposure times, are shown in Fig. 2. In DAOY cells, the signals at 0.9, 1.3, 2.8 and 5.4 ppm start to increase by 24 h and continue to increase with further treatment to 48 h. In PFSK-1 no change in ^1^H NMR lipid peaks was detected.

**Fig. 3:**
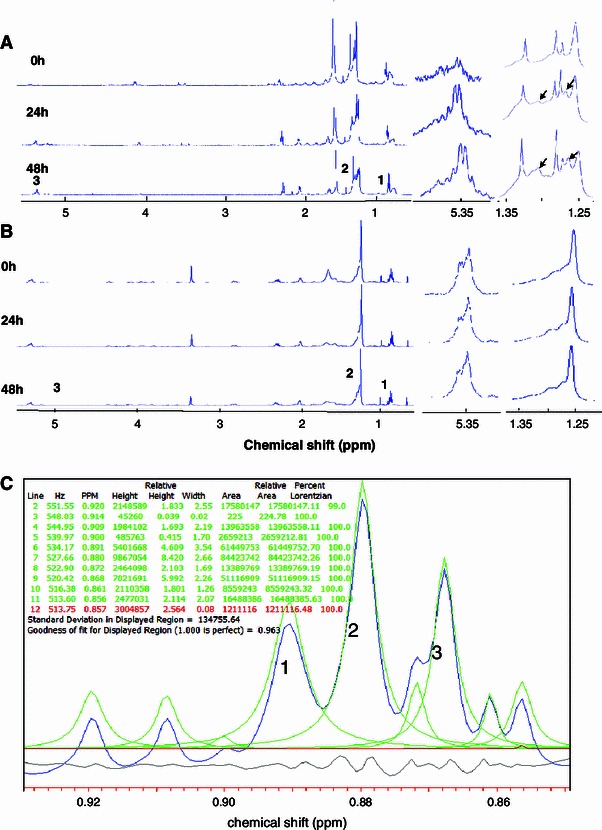
^1^H NMR spectra of lipid extracts from **a** isolated LDs of DAOY cells and **b** whole DAOY cells treated with 10 μM cisplatin at 0, 24 and 48 h. The spectra are scaled such that the CH_3_ triplet is the same intensity. Assignments are as follows: 1 C**H**_3_ at 0.9 ppm, 2 C**H**_2_ at 1.2–1.3 ppm, 3 **H**C=C**H** (L, Ch) at 5.4 ppm. To the right is the 5.4 ppm region and 1.3 ppm region expanded to show the lipid signal from **H**C=C**H** and C**H**_2_ (indicated by *arrows*), respectively. The intensities of these peaks have been increased by the same arbitrary factor for all timepoints. (**c**): Line fit for the –CH_3_ triplet (labelled as 1, 2 and 3) signal at 0.9 ppm

Figure 3a shows ^1^H NMR spectra of lipid species in extracts from isolated LDs following cisplatin treatment of DAOY cells. For comparison, Fig. 3b shows the ^1^H NMR spectra of lipid species in extracts from whole DAOY cells following cisplatin treatment. The spectra over the range 1–5 ppm in Fig. 3a, b are scaled to give the same intensity of the CH_3_ resonance. An expansion of the 5.35 ppm and 1.25–1.35 ppm regions is provided to show the increase of CH=CH and –CH_2_ signal (arrows in Fig. 3a) from unsaturated lipids. For each expanded region, the intensity of the spectra has also been increased by the same arbitrary factor for all time points in order for them to be large enough to appreciate the differences in intensity within a time series. The fitting of the spectral region containing the CH_3_ resonance is shown in Fig. 3c.

**Fig. 4:**
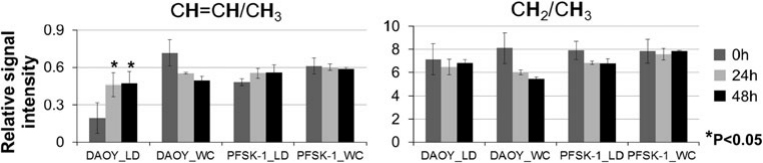
Double bond proton signal/methyl group proton signal (CH=CH/CH_3_) and methylene group proton signal/methyl group proton signal (CH_2_/CH_3_) from LD lipids and whole cell (*WC*) lipids of DAOY and PFSK-1 cell extract spectra

Quantification of the ^1^H NMR spectra acquired from lipid extracts showed that there was a statistically significant increase in the CH=CH/CH_3_ ratio in lipids in LDs from cisplatin treated DAOY cells (Fig. 4). No such change was observed in LDs from PFSK-1 (Fig. 4). In whole cell lipid extracts, the CH_2_/CH_3_ and CH=CH/CH_3_ ratios remained unchanged by treatment for both cell lines, the apparent decrease in the DAOY cell line was not statistically significant. One should note that in untreated DAOY cells the ratio of CH=CH/CH_3_ of LD lipids was much lower than in PFSK-1, possibly due to inherent properties of the cell types, but these ratios were similar in lipids extracted from whole cells (including membrane associated lipids as well as lipid droplets).

**Fig. 5:**
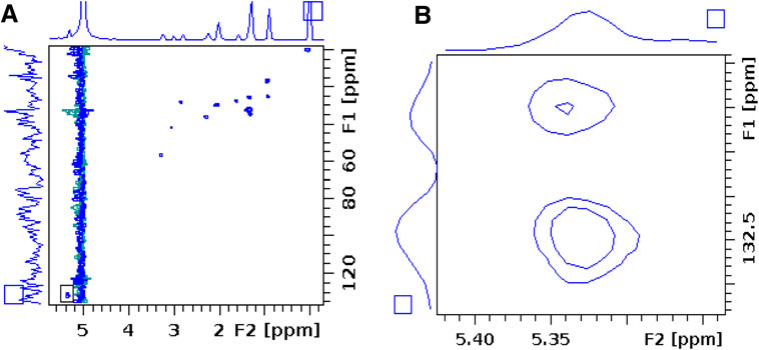
HSQC spectra of 48 h 10 μM cisplatin treated DAOY **a** whole spectrum from 0 to 6 ppm (^1^H) and 0 to 140 ppm (^13^C), **b** expanded 5.44 to 5.35 ppm region of the spectrum

The HSQC (^1^H and ^13^C) spectrum acquired from 48 h cisplatin treated DAOY cells is shown in Fig. 5. The chemical nature of unsaturated lipids is of special interest (Griffin et al. 2003; Pan et al. 2012) but cannot be determined specifically from the 1D NMR spectra. The double bond protons of unsaturated fatty acids resonating at 5.4 ppm region in the 1D spectra were separated into two distinguishable signals by the 2D HSQC assigned to oleic (18:1) and linoleic acid (18:2) (Pan et al. 2012). The double bond protons in linoleic acid give two signals with an equal intensity at the 5.4 ppm making it possible to estimate the ratio of these two unsaturated fatty acids. An oleic-to-linoleic acid ratio was found to be 1:0.89 in cisplatin treated DAOY cells.

**Fig. 6:**
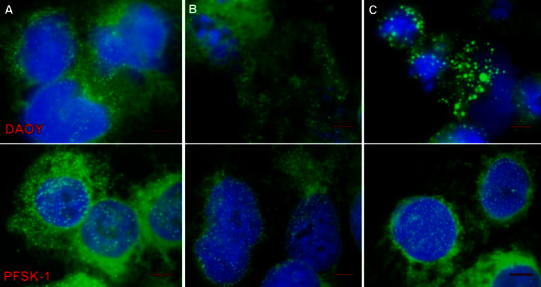
Combined Nile red and DAPI staining of DAOY (*upper*) and PFSK-1 (*lower*): **a** untreated cells at 0 h, **b** 10 μM cisplatin 24 h, **c** 10 μM cisplatin 48 h. The size *bar* is 5 μm

LDs are visualised as green vesicles in Nile red stained cells, while cell nuclei stained with DAPI appear blue (Fig. 6). There was an increase in the number of small LDs (approximate diameter of 0.2 μm) by 12 h of 10 μM cisplatin treatment in DAOY cells, while nuclei remained intact, with a diameter around 15–20 μm. A ring-like arrangement of small LDs was evident in DAOY cells when the LD size increased by 24 h of cisplatin treatment. After 48 h treatment, large LDs were seen with concomitant fragmentation of nuclei. The average diameter of LDs of 0.18 ± 0.06 μm in untreated DAOY cells remained unchanged until 24 h of cisplatin exposure, but it had increased to 0.34 ± 0.19 μm by 48 h (*P* < 0.001 Student *t* test). There was no change in either the number or the size of LDs in PFSK-1 cells exposed to 10 μM cisplatin, similar to untreated DAOY cells.

## Discussion

The current study shows that unsaturated fatty acids detected by HR-MAS increase in whole DAOY cells undergoing cisplatin-induced cell death, consistent with studies on a glial tumour (Hakumaki et al. 1999) and lymphoma cells (Schmitz et al. 2005). Extracts of LDs show an increase in unsaturated fatty acids which reflect those seen in HR-MAS of whole DAOY cells undergoing apoptosis consistent with the ^1^H NMR signal arising from the lipids pool present in LDs. The latter observation is in agreement with previous cell (Quintero et al. 2007; Zietkowski et al. 2012) and brain tumour (Griffin et al. 2003; Hakumaki et al. 1999) studies. These changes in lipid composition are not detected in lipid extracts from whole cells showing that the lipids from non-LD sources (such as membranes) dilute observations from a specific pool of ^1^H NMR visible species. It was recently shown that apoptosis in EL4 lymphoma cells is accompanied with increased incorporation of external oleate into triacylglycerols (Boren and Brindle 2012). Considering the ratio of oleic-to-linoleic acid in LDs from cisplatin treated DAOY cells, it is possible that increase in unsaturation can be ascribed to excessive accumulation of linoleic acid and its incorporation into triacylglycerols. Two mechanisms have been proposed to contribute to the increased unsaturated ^1^H NMR detectable lipids in apoptotic cells: (a) repartitioning from the cellular membrane degradation (Griffin et al. 2003) and (b) de novo synthesis of lipids. (Boren and Brindle 2012; Zietkowski et al. 2012).

Cisplatin as a chemotherapy drug has been used to treat various types of cancers including childhood medulloblastoma (Jakacki et al. 2012; Kim et al. 2012; Shulman et al. 2012). It causes crosslinking of DNA, which leads to tumour cell apoptosis (Tanida et al. 2012). Here DAOY cells derived from childhood medulloblastoma show greater sensitivity to cisplatin treatment than PFSK-1 cells. Cellular nuclear fragmentation of treated DAOY cells was observed as a sign of DNA damage.

LD accumulation is a common feature frequently observed in tumour cells (Delikatny et al. 2002) and tissues (Opstad et al. 2008) undergoing apoptosis and necrosis. DAOY and PFSK-1 are both primitive neuroectodermal tumour cell lines possessing a similar number and size of LDs without treatment. LDs accumulate in DAOY cells undergoing apoptosis and LDs up to 1.67 μm in diameter were detected after 48 h cisplatin treatment. No obvious increase in LD size was observed in PFSK-1 cells with intact nuclei (Fig. 6). The increase of unsaturated fatty acids (UFAs) detected by ^1^H NMR in treated DAOY cells was not observed in PFSK-1 cells. These observations imply that UFAs accumulate in LDs in response to treatment. We observed greater number of small LDs in DAOY cells at 12 h of cisplatin exposure, but this increase was not large enough to result in increase in ^1^H NMR detected lipids in HR-MAS spectra most likely due to the fact that increase in overall volume of NMR detectable lipids was below the limit of detection. At later stages of apoptosis large LDs appear, possibly reflecting shunting of lipids released through lysosomal processing of lipids from damaged cell membranes and organelles (Delikatny et al. 2002). A recent study by Boren and Brindle on lymphoma cells treated with etoposide showed that β-oxidation becomes inhibited early on in apoptosis leading to increased de novo synthesis of lipids and accumulation of LDs. Among UFAs, PUFAs are thought to be naturally occurring anti-cancer agents (Cockbain et al. 2012). In human glioma, PUFA may stimulate tumour regression and apoptosis (Scheim 2009) and has been introduced to cancer therapy (Bakshi et al. 2003). It is not known yet whether any of these lipid-based approaches to cancer therapy will show selectivity for malignant cells, but PUFA supplementation may potentiate some anti-cancer treatment strategies (Burns and Spector 1994). Enrichment with PUFAs makes cancer cells more susceptible to lipid peroxidation and more sensitive to drug treatment (Burns and Spector 1994). In addition, the immune response in cancer can be improved by manipulating the lipid levels in dendritic cells (Herber et al. 2010). Therefore, accumulated PUFAs after cisplatin exposure may not only be end products of cell collapse but they may also serve as functional lipid mediators actively involved in the cell death pathway.

## Concluding remarks

HR-MRS of whole DAOY cells detects an increase in lipids and the proportion of these lipids which are unsaturated during treatment with cisplatin. These changes are associated with an increase in the number of cytoplasmic LDs and these droplets contain a greater proportion of unsaturated lipids than in untreated cells. An increase in the proportion of unsaturated fatty acids is not seen in the lipids extracted from whole cells during treatment. Our data argue that it is pivotal to pay attention to the degree of lipid unsaturation when considering ^1^H MRS in monitoring treatment response.
